# Is an Appreciation of Isomerism the Key to Unlocking the Mysteries of the Cardiac Findings in Heterotaxy?

**DOI:** 10.3390/jcdd5010011

**Published:** 2018-02-06

**Authors:** Robert H. Anderson, Diane E. Spicer, Rohit Loomba

**Affiliations:** 1 Institute of Genetic Medicine, Newcastle University, Newcastle upon Tyne NE1 3BZ, UK; 2 Division of Pediatric Cardiology, University of Florida, Gainesville, FL 32610, USA; spicerpath@hotmail.com; 3 Division of Pediatric Cardiology, Advocate Children’s Hospital, Oak Lawn, IL 60453, USA; r.loomba@gmail.com

**Keywords:** situs ambiguus, right isomerism, left isomerism, dextrocardia, ventricular looping, ventricular topology, sequential segmental analysis

## Abstract

Pediatric cardiologists treating patients with severe congenital cardiac defects define “visceral heterotaxy” on the basis of isomerism of the atrial appendages. The isomeric features represent an obvious manifestation of disruption of left-right asymmetry during embryonic development. Thus, there are two subsets of individuals within the overall syndrome, with features of either right or left isomerism. Within the heart, it is only the atrial appendages that are truly isomeric. The remainder of the cardiac components shows variable morphology, as does the arrangement of the remaining body organs. Order is provided in this potentially chaotic arrangement simply by describing the specific features of each of the systems. These features as defined by clinicians, however, seem less well recognized by those investigating the developmental origins of the disruption of symmetry. Developmental biologists place much greater emphasis on ventricular looping. Although the direction of the loop can certainly be interpreted as representing an example of asymmetry, it is not comparable to the isomeric features that underscore the clinical syndromes. This is because, thus far, there is no evidence of ventricular isomerism, with the ventricles distinguished one from the other on the basis of their disparate anatomical features. In similar fashion, some consider transposition to represent abnormal lateralization, but again, clinical diagnosis depends on recognition of the lateralized features. In this review, therefore, we discuss the key questions that currently underscore the mismatch in the approaches to “lateralization” as taken by clinicians and developmental biologists.

## 1. Introduction

Many features of the congenitally malformed heart remain difficult to understand and interpret to those who are unfamiliar with the lexicon of pediatric cardiologists. This is particularly true with regard to the developmental background of the condition described by many pediatric cardiologists as “visceral heterotaxy”. The lesions labelled in this fashion by the pediatric cardiologists account for the most complex combinations of congenital cardiac malformations [1]. Within these complex combination of lesions, nonetheless, it is well recognized that there are two subsets of patients, with markedly different features [2,3]. Despite these marked differences in morphological features of the two subsets, however, it has thus far been usual for those seeking to unravel the genetic background of the syndromes to group together patients when searching for potential genetic cues. This is tantamount to mixing apples with oranges. The two subsets were initially subdivided on the basis of splenic morphology [2,3]. It is now established that, from the stance of cardiac morphology, the essential differences between the patients within the groups is the presence of isomeric rather than lateralized atrial appendages [4,5]. Such categorization on the basis of left rather than right isomerism of the atrial appendages provides the necessary specificity to appreciate the markedly variable features to be found within the heart, and also to provide correlation with the arrangement of the other thoraco-abdominal organs [6,7]. The presence of isomerism within the atrial component of the heart unequivocally points to an obvious deficiency of left-right patterning during embryonic development. As yet, however, the genetic problems underscoring the finding, in the clinical setting, of patients with isomeric rather than lateralized atrial appendages has yet to be unraveled. At the same time, huge strides have been made by developmental biologists in showing the pathways that lead to establishment of the left as opposed to right sides of the individual [8,9]. It would seem, nonetheless, that the developmental biologists take a different approach to the identification of “lateralization”. It is also the case that developmental biologists continue to interpret heterotaxy on the basis of the original approach taken by Geoffroy St Hilaire, namely any arrangement of the organs that is other than normal. There is much to commend this approach, not least because it maintains the vernacular definition of heterotaxy. For better or worse, the International Nomenclature Committee responsible for providing definitions of congenital cardiac lesions have restricted “heterotaxy” to the lesions characterized by the presence of isomerism of the atrial appendages [10]. These differences in the approach to symmetry and lateralisation have resulted in a current disconnect in the searches for the answers to the problems producing the clinical syndromes of isomerism. In this review, therefore, our aim is to emphasise the features that underscore the existence of cardiac isomerism, namely the isomeric features of the atrial appendages. When analysis begins with attention to the atrial appendages [11], it proves possible to establish a logical approach which provides the understanding of the cardiac features of the subsets currently identified by many pediatric cardiologists as representing “heterotaxy”. We aim to show that specific description of the features of the remaining systems of organs removes any suggestion that the syndromes are those producing “situs ambiguus” [3]. We begin, nonetheless, by discussing the fundamental anatomical differences between the types of lateralization represented by ventricular looping [12,13] as opposed to isomerism of the atrial appendages.

## 2. What Is “lateralization” and “Breaking of Symmetry”?

**Figure 1:**
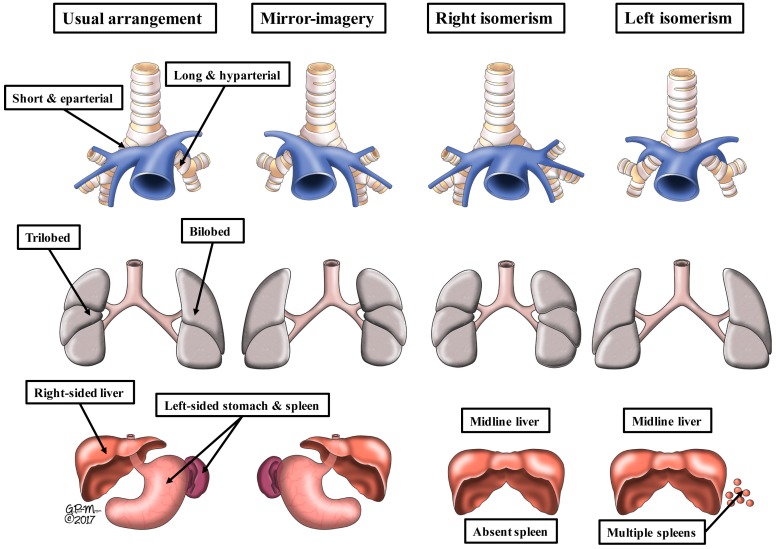
The drawings show the variations of the bodily organs in the lateralized (left-hand two columns) and isomeric (right-hand two columns) arrangements as seen in the clinical setting. The upper panels show the bronchial morphology, and the relations of the bronchuses to the pulmonary artery feeding the lower lobes of the lungs. The middle panels show pulmonary morphology. Note that the artist has emphasized the differences in lobation, but in so doing, has distorted the arrangement as seen during life. The lobes are shown as seen from the side, even though illustrated as if seen from the front. The lower panels show the arrangement of the liver, stomach, and spleen. The stomach can either be right-sided or left-sided in the isomeric patterns. Multiple spleens exist only when splenic tissue is present on both sided of the mesogatrium. Although usually harmonious, the arrangement of the abdominal organs is not always in keeping with pulmonary and bronchial arrangements. For this reason, analysis of cardiac findings should start with knowledge of the arrangement of the atrial appendages (see Figure 2).

Elegant and extensive reviews are now available to account for the molecular and genetic developmental changes that underscore the establishment of the basic building plan of the individual. During development, a key step in these changes is the establishment of the morphological left, as opposed to the morphologically right, sides of the body. These differences, involve only the visceral components, since all individual mammalians show isomerism of their parietal structures, with the left-sided skeletal muscles, for example, being mirror-images of the right-sided muscles. Extending this analogy, in the human individual the hands can be taken as the exemplar of bodily isomerism. In such normal individuals, the lungs and the bronchial tree can be taken as examples of structures that are lateralized (Figure 1-left-hand panel).

There is also evidence of lateralization within the heart, with both the morphologically right atrial and ventricular chambers being distinguished from their morphologically left partners because of their anatomic differences. Such anatomic lateralization, however, is also seen when the heart itself is right-sided as opposed to left-sided, and when its apex points to the right, rather than the left. This means that the topological arrangement of the ventricular mass, for example, cannot necessarily be taken as evidence of “lateralization” when considered relative to the concept of cardiac isomerism. In the lesion known as congenitally corrected transposition, the essential feature is that the topology of the ventricular mass does not match the arrangement of the atrial chambers. This is described as representing discordant atrioventricular connections. It can be found irrespective of the direction of cardiac looping, and irrespective of the location of the heart within the body, or the direction of the cardiac apex. For the pediatric cardiologist, therefore, as we will discuss in greater detail below, there is a fundamental difference between lateralization of the ventricular chambers and isomerism of the atrial appendages. One of the recent erudite discussions on “breaking symmetry”, when seeking to relate this to morphogenesis [9], concentrated exclusively on ventricular looping when considering cardiac development. No mention was made of isomerism of the atrial appendages. Another remarkably well referenced review, published in this journal, noted that “looping is an intrinsic property”, but then made no attempt to distinguish the differences between the establishment of left versus right isomerism [8]. This is the more surprising, since in an earlier investigation, these authors had explored the differences between the establishment of isomerism within the atrial as opposed to the ventricular components of the developing heart [14]. In this earlier study, they pointed to the key influences of genes such as Pitx2c during early development, but then established that these influence were lost subsequent to looping. They emphasized that the original differences between morphologically rightness and leftness were maintained in the atrial chambers, but lost in the ventricles. On this basis, they suggested that the ventricles might better be considered systemic and pulmonary, rather than “right” and “left”. This suggestion, however, founders when assessed in the setting of congenitally corrected transposition, where it is the morphologically left ventricle that supports the pulmonary circulation. The authors were also mistaken when they supposed that the ventricles might be affected in the clinical syndromes of isomerism. As we will discuss, there is no evidence of ventricular isomerism in the patients now diagnosed because of the isomerism of their atrial appendages. It is, nonetheless, the morphologically leftness as opposed to rightness of the appendages that serves to differentiate the isomeric syndromes. Moreover, paradoxically, it was the demonstration of unequivocal isomerism of the appendages in knock-out mice [15,16] that served to dispel the notion that there was no such thing as cardiac isomerism [17]. When relating developmental events to the cardiac syndromes identified on the basis of the presence of isomeric atrial appendages, therefore, it is crucial to distinguish between the atrial features and ventricular morphology. Indeed, it is now established that, so as properly to describe the features when each atrium is joined to its own ventricle, it is necessary to describe both the type of isomerism and the topology of the ventricular loop [5,11]. In this way, it is possible to distinguish between the mixed atrioventricular connections, dispelling the notion that they might be “ambiguous”, which had been our earlier approach to description. It is also crucial, if we are to avoid misunderstanding, to emphasise the different approaches taken by many pediatric cardiologists as opposed to development biologists with the use of “heterotaxy”.

## 3. What Is Heterotaxy?

When defined literally, “heterotaxy” is any departure from the normal arrangement. In this regard, we now know that there are four basic patterns of bodily arrangement. The usual arrangement, found in the overwhelming majority of individuals, is usually called “situs solitus” by pediatric cardiologists. In this arrangement, the thoraco-abdominal organs are lateralized, differing in the anatomical features of their right and left sides (Figure 1-left-hand panel). The second pattern, which again shows the features of lateralization of the thoraco-abdominal organs, is mirror-imagery, usually described as “situs inversus” (Figure 1-second left-hand panel). Individuals with mirror-imagery make up the smallest group of those with unexpected bodily arrangements. Many pediatric cardiologists, particularly those working in North America, currently exclude their patients with mirror-imagery, or “situs inversus”, from the category of “visceral heterotaxy”. There is no question but that mirror-imagery represents a departure from the normal. It is, therefore, unfortunate that the members of the International Nomenclature Society chose to exclude mirror-imagery when defining “heterotaxy” [10]. This was because, at the time, there remained doubt with regard to the existence of isomeric features within the heart [3]. This, in turn, was because the cardiac features had been described in terms of “atrial isomerism” [17]. The argument had been made that, if there was true “atrial isomerism”, patients with the left variant would have eight pulmonary veins. This approach, of course, reduces the situation to the absurd. It was subsequently shown, by concentration on the extent of the pectinate muscles within the atrial appendages relative to the atrioventricular junctions [6], that cardiac isomerism was a real thing, permitting immediate distinction from the arrangements found when the atrial appendages were lateralized. The key finding was that the isomeric features involved only the atrial appendages [6]. Moreover, as already discussed, genetic manipulation of mice has now confirmed that the appendages can be found with the features of either left or right isomerism [15,16]. It is the recognition that only the atrial appendages are truly isomeric in the setting of the syndromes described by pediatric cardiologists as “visceral heterotaxy”, as we will show, that provides the basis for logical description of the cardiac findings in the two known clinical subsets. This then permits appropriate correlation with the remaining systems of organs.

## 4. What Is Isomerism?

**Figure 2:**
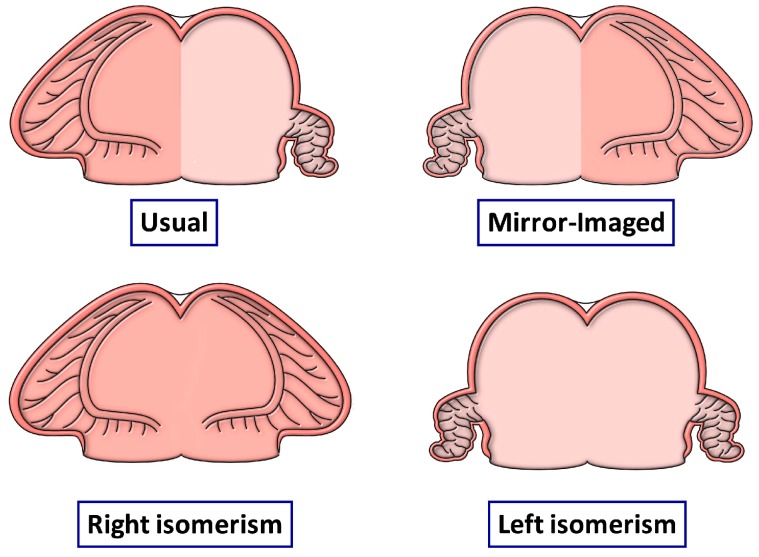
The drawings show the possible arrangement of the atrial appendages, as judged on the basis of the extent of the pectinate muscles within the appendages relative to the atrioventricular junctions. Although the arrangement of the appendages is usually in harmony with the arrangement of the other thoraco-abdominal organs, as shown in Figure 1, this is not always the case. For this reason, analysis of cardiac arrangement should start with the arrangement of the atrial appendages. Any disharmony with the remaining organs should then be noted.

We have already discussed how the arrangement known as “situs inversus” is the mirror-imaged variant of the usual bodily arrangement. In the “inversus” pattern, all the organs are on the “wrong” side of the body (Figure 1-second left-hand panel). In individuals with either the usual arrangement of the bodily organs (Figure 1-left-hand panel), however, or in those with its lateralized but mirror-imaged variant, also as we have already discussed, it is only the organs themselves that are lateralized. The parietal components of the body, such as the limbs and the sides of the head and trunk, are themselves mirror-images of each other, but in the same individual. This mirror-imagery within the same individual of the visceral structures is the essence of bodily isomerism (Figure 1-right-hand two panels). The arrangements shown in Figure 1 take account only of the patterns of the lungs, the bronchuses supplying them, and the abdominal organs. As can be seen, it is only the lungs and bronchuses that are truly isomeric. Although there is usually harmony between the arrangement of the pulmonary and bronchial arrangements with the abdominal organs, particularly the spleen, this is not always the case. It was for this reason that, for quite some time, the isomeric arrangements were considered to represent “situs ambiguus” [2]. It is now recognized that any potential ambiguity is readily removed simply by describing the arrangement of each set of organs, in this way drawing attention to any disharmonious combinations. It is because of the finding of such disharmony that it is essential to describe findings within the heart on the basis of the specific arrangement of the cardiac segments. The starting point for cardiac analysis is the morphology of the atrial segment [3]. When assessed according to the extent of the pectinate muscles within the atrial appendages relative to the atrioventricular junctions, there are only four possible atrial arrangements [6]. These are the usual arrangement, when the pectinate muscles encircle the right-sided atrioventricular junction, but are confined within the tubular appendage on the left side, the mirror-imaged variant, or the variants with either isomeric right or left atrial appendages (Figure 2).

The arrangement of the atrial appendages is usually congruent with the bronchial morphology [7], but this is not always the case. Harmony with the splenic arrangement is less uniform than with the bronchial patterns [5,11]. In the past, when the cardiac findings in so-called heterotaxy were usually segregated on the basis of splenic morphology [2,3], this led to the somewhat ridiculous situation of description of presence of a spleen in the “asplenia” syndrome. These potential problems are removed when analysis starts with description of atrial arrangement, taking care to describe any disharmonies with either the broncho-pulmonary patterns or the arrangement of the abdominal organs [11]. The arrangement of the atrial appendages is usually congruent with the bronchial morphology [7], but this is not always the case. Harmony with the splenic arrangement is less uniform than with the bronchial patterns [5,13]. In the past, when the cardiac findings in so-called heterotaxy were usually segregated on the basis of splenic morphology [2,3], this led to the somewhat ridiculous situation of description of presence of a spleen in the “asplenia” syndrome. These potential problems are removed when analysis starts with description of atrial arrangement, taking care to describe any disharmonies with either the broncho-pulmonary patterns or the arrangement of the abdominal organs [12].

## 5. What Is Isomeric within the Heart?

**Figure 3:**
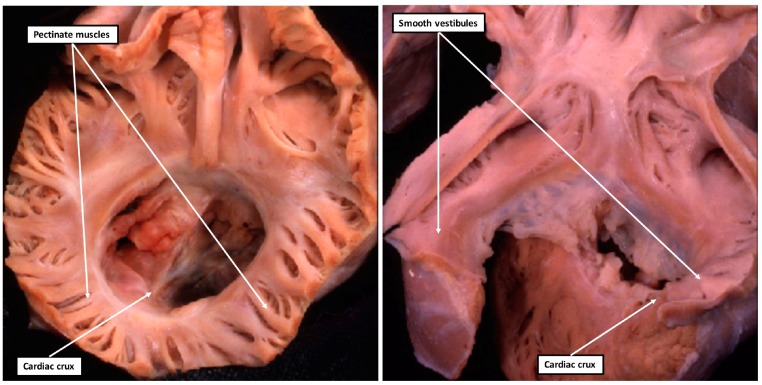
The images show the features of isomeric right (left-hand panel) and isomeric left (right-hand panel) atrial appendages. In both hearts, the atrioventricular junctions are viewed from above and from behind. The left-hand panel shows the pectinate muscles extending bilaterally to meet at the cardiac crux, while the caudal parts of the atrial vestibules are smooth in the heart shown in the right-hand panel, with left isomerism. Both hearts also have a common atrioventricular junction guarded by a common valve separated into right and left ventricular orifices by the presence of a tongue of valvar tissue joining together the leaflets that bridge the ventricular septum.

As has already been emphasized, one of the problems with the first suggestion that isomerism was to be found within the heart was that the findings were described in terms of “atrial isomerism” [17]. Subsequent investigations showed that, when assessed according to the arrangement of the pectinate muscles, as shown in Figure 2, then it was only the appendages that were truly isomeric [5,11]. This finding in itself then means that it is essential fully to describe the remainder of the cardiac findings. This is now readily achieved using the system known as sequential segmental analysis [18]. The system is as valuable when analyzing genetically modified mice as it has proved to be in the clinical setting. The key to establishing the presence of isomerism, as we have already stressed several times, is the extent of the pectinate muscles relative to the atrioventricular junctions. The finding of right isomerism is particularly obvious when based on this feature (Figure 3-left hand panel). The finding of left isomerism is not quite as clear cut, particularly when analyzing morphological findings in autopsy specimens that have often been the subject of previous dissections, but the lack of pectinate muscles extending to the crux on either side is significant (Figure 3-right hand panel).

Having diagnosed the presence of isomeric right as opposed to left atrial appendages, certain features of the intracardiac anatomy then cluster with right as opposed to left isomerism. The coronary sinus is universally absent in the setting of right isomerism. The coronary sinus can also be absent when both appendages are of left morphology, but more frequently there is either a right- or left-sided superior caval vein draining along the atrioventricular junction in the fashion of a morphologically left caval vein. The pulmonary veins are frequently connected in bilateral fashion in the setting of isomeric left appendages, but all four pulmonary veins can connect to either the left- or the right-sided atrial chamber. The pulmonary veins, of necessity, are connected in anomalous fashion with isomeric right appendages, even if coming back to the heart. In around half of patients with right isomerism, nonetheless, the pulmonary veins drain anomalously to an extracardiac site. It is interruption of the inferior caval vein that is the anticipated finding in the setting of left isomerism, again not an unexpected finding since the inferior caval vein itself is a morphologically right-sided structure. On occasion, however, the venoatrial connections can be arranged in such a way as to produce quasi-usual or quasi-mirror-imaged venous returns. This does not detract from the fact that the atrial appendages themselves are isomeric. Patients with both types of isomerism can have bilateral superior caval veins, but only in the setting of left isomerism is it possible for one or other of the superior caval veins to drain to the heart through a coronary sinus. Common atrioventricular junctions are also frequent findings in both types of isomerism (Figure 3), although only in the setting of right isomerism is it frequent to find the atrioventricular junction connected predominantly to only one ventricle, often the morphologically right ventricle. Discordant or double outlet ventriculo-arterial connections are much more frequent in the setting of right isomerism, but can also be found with left isomerism, although the ventriculo-arterial connections are much more frequently concordant in the latter setting. Left-handed as opposed to right-handed topology can be found with either type of isomerism, and neither the sidedness of the heart nor the direction of its apex discriminates between the isomeric variants. True ventricular isomerism has yet to be found in the examination of large numbers of hearts from patients with right and left isomerism [5,6]. Obstruction or atresia of the right ventricular outflow tract, however, is a feature of right isomerism, while left ventricular outflow tract obstruction, along with aortic coarctation and interruption, is more frequent with left isomerism. Perhaps surprisingly, it is exceedingly rare to find symmetrical formation of the aortic arches in either right or left isomerism, as in the setting of so-called vascular rings. The sidedness of the aortic arch itself also fails to discriminate between the subsets. These findings do no more than emphasise that the attention of the morphologist, when seeking to distinguish between the isomeric subsets of heterotaxy, should be directed at the atrial chambers, and in particular on the extent of the pectinate muscles lining the atrial appendages (Figure 3). This will then also serve to distinguish the isomeric variants from those having mirror-imagery. This is of significance since, in the past, patients with isomeric appendages were often considered to show “partial situs inversus”. In reality, patients with isomerism are as different from those with mirror-imagery as from those with normally arranged organs.

## 6. How Do the Morphological Findings Relate to Cardiac Development?

**Figure 4:**
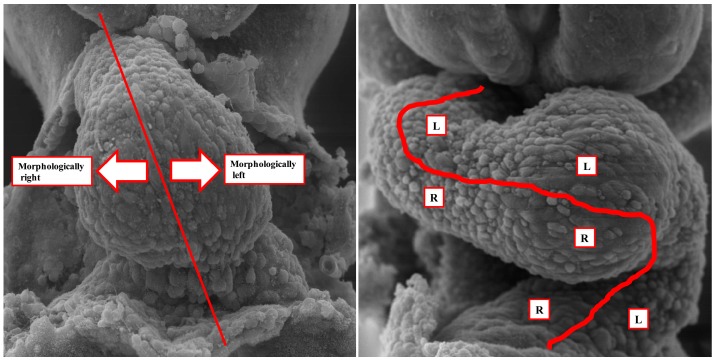
The images show the steps in formation of the ventricular loop in the mouse heart at around embryonic day 8.5 to 9.5. The left-hand panel shows the stage at which material is being added at the arterial and venous poles from the second heart field. The initial linear heart tube, with its right and left sides, will form little more than the definitive left ventricle; The right-hand panel shows the situation subsequent to formation of the ventricular loop. The atrial appendages will balloon in parallel from the right and left sides of the atrial component of the initial heart tube, and hence respond in different fashion to the genes responsible for producing morphologically leftness as opposed to rightness. These genes, however, will influence both ventricles in comparable fashion.

It is becoming increasingly frequent for developmental biologists to seek abnormalities in left-right patterning when assessing the findings in genetically manipulated mice. As we have emphasized, it was the production of genetically engineered mice that proved the existence of cardiac isomerism [15,16]. All too often, however, developmental biologists fail to distinguish between the mechanisms of ventricular looping and those responsible for production of isomeric atrial appendages. We have commented on the failure to discuss the features of the atrial chambers in a recent excellent review of the molecular background to left-right patterning [9]. A recent experimental study of a gene new to us similarly failed to assess the atrial chambers when considering left-right symmetry relative to congenital cardiac malformations [12]. By failing to assess the morphology of the atrial chambers, such investigators rule out perhaps the most important indicator of abnormal laterality, at least as assessed by the pediatric cardiologist. Attention to ventricular topology fails to discriminate the clinical subsets. As already discussed, patients with either subset of isomerism can be found with either right-handed or left-handed ventricular topology. It is the left-handed variant that is usually interpreted as being dependent on leftward looping of the developing ventricles. It is pertinent, therefore, to consider the steps involved in formation of the ventricular chambers during normal development. In early stages of development, a linear heart tube can be recognized within the developing pericardial cavity (Figure 4-left-hand panel). This component, however, subsequently forms little more than the definitive left ventricle. It is subsequent to addition of new material from the second heart field that it becomes possible to recognize the ventricular loop (Figure 4-right-hand panel).

The trabecular components of the ventricles, which permit their distinction in the postnatal heart, then balloon from the inlet and outlet components of the loop in series. The atrial appendages, in contrast, balloon from the common atrial component of the heart tube in parallel. It is in consequence of these morphogenetic events, therefore, that the right and left sides of the atrial component of the heart are able to respond to the genetic cues determining morphologically rightness and leftness, respectively. These cues, in contrast, affect both halves of the developing ventricles in comparable fashion. These features had all been emphasized in the experimental study conducted by Campione and colleagues [14]. As they rightly commented when considering the effects of Pitx2, “atrial and ventricular expressions are independent”. The focus of those seeking the influence of disturbed left-right patterning, therefore, should surely be directed to the atrial components of the heart, rather than the ventricular loop. In particular, those seeking evidence of laterality disturbances should be focusing their attention on the extent of the pectinate muscles within the atrial appendages, and the arrangement of the venoatrial connections. These are just as pertinent for those analyzing changes in morphology of the mouse heart as for the human heart (Figure 3).

## 7. Does “Dextrocardia” Have Any Meaning When the Heart is Congenitally Malformed?

The notion of an abnormal formation of the heart loop representing an indicator of a disturbance in laterality may then be translated into the interpretations of an “inverted” heart as evidence of the presumed disturbance [12]. When investigators describe an “inverted” heart in this context, it usually means that the heart is located in the right rather than the left chest, with its apex pointing to the right. Many pediatric cardiologists also still describe this arrangement, when seen in the clinical setting, as “dextrocardia”. One of the earliest investigations establishing the importance of segmental analysis, nonetheless, showed that the presence of the heart in the right chest conveyed no information with regard to the make-up and connections of its contained components [19]. There are many reasons why the heart can be located in the right chest, such as diaphragmatic hernia, with a space-occupying lesion pushing the heart to the right. In patients with mirror-imaged arrangement of the bodily organs, furthermore, a right-sided heart, with its apex pointing to the right, is a “normal” finding. This is not to deny that the heart can be abnormally located in patients having congenitally malformed hearts. Such a finding, however, does no more than to emphasize the need for further investigations so as to establish the sequential segmental arrangements present. When the atrial appendages are isomeric, nonetheless, the sidedness of the heart conveys no specific information regarding the type of isomerism, nor the specific ventricular topology.

## 8. Is “Transposition” a Disturbance of Laterality?

In their recent study, the group working in Monash [12] interpreted the origin of both arterial trunks arising from the right ventricle as representing a disturbance of laterality. This is somewhat surprising, since in early development it is the norm for the undivided proximal outflow tract to be supported exclusively by the developing right ventricle. Retention of part of normal development, therefore, should hardly be considered as a disturbance of laterality, at least when laterality is considered an antonym to isomerism. There are, furthermore, several variants of double outlet right ventricle as seen in the clinical setting. One of these variants is related to transposition, better described in terms of discordant ventriculo-arterial connections. Transposition is also interpreted by some as representing a defect in lateralization. It is certainly the case that discordant ventriculo-arterial connections, or “transposition”, and for that matter double outlet right ventricle, can be found with some frequency in patients with both types of isomerism, but particularly right isomerism. When account is taken of the overall number of patients encountered in the clinical setting with discordant ventriculo-arterial connections as their principal lesion, however, bodily isomerism as opposed to usual or mirror-imaged atrial arrangement is found in a very small minority. If transposition was, indeed, a disturbance of laterality we would anticipate a far greater proportion of those with discordant ventriculo-arterial connections to possess isomeric atrial appendages. It is our opinion that transposition is better considered as a problem in segmental connections, rather than lateralization. Moreover, properly to determine the genetic cues leading to isomerism, as opposed to lateralized bodily arrangement, we suggest it is likely to be helpful to assess separately the findings in patients having transposition according to the specific arrangements of their atrial appendages. We suggest that the same concept should now apply equally for those investigating genetically modified mice, where the information available from episcopic analysis of the malformed hearts readily permits the precise identification of atrial morphology.

## 9. Discussion

The precise mechanism underpinning the production of the features of isomerism as opposed to lateralization of the visceral organs, despite the huge strides made in establishing the steps involved in left-right determination [8,9], has yet to be identified. Over the period in which the biologists have been exploring the molecular and genetic changes, those working in the clinical arena have made similar strides in refining the systems used for the description and categorization of congenitally malformed hearts. The conflicts that initially existed in terms of the systems used for classification were resolved by the establishment of an International Nomenclature Committee. This Committee produced a cross-map of the various names of different lesions, and has now provided definitions that will be incorporated in the eleventh iteration of the International Classification of Disease produced by the World Health Organisation [20]. It is, perhaps, unfortunate that the definition for heterotaxy is at variance with that used by developmental biologists. Despite such ongoing differences, the lessons learned by those working in the clinical field should be of value to those investigating the cardiac malformations encountered in genetically modified mice. Failure to take note of the morphology of the lesions as encountered in patients when describing mouse malformations has the potential to create considerable confusion. An example is the recent claim by the group working in Pittsburgh that they had created mouse models for “hypoplastic left heart syndrome” [21]. In reality, none of their mice had the features recognized in humans as representing hypoplastic left heart syndrome, which is found in the setting of an intact ventricular septum and concordant ventriculo-arterial connections [22]. Similar caveats are pertinent to those investigating the genetic background to so-called disturbances of laterality. Much has been learned over the same decades as clinical pediatric cardiologists have resolved their differences in terminology to link the laterality disturbances to early stages of embryonic development. The evolution of understanding was well reviewed in the recent investigation reported by the group working in Monash [12]. Knowledge of the comparable advances made in the understanding of congenital cardiac malformations will surely unlock the secrets of formation of an isomeric arrangement of the thoracic organs, as opposed to the usual or mirror-imaged patterns seen in the majority of individuals. The bottom line is that it is the arrangement of the atrial appendages that indicates the presence of cardiac isomerism, as opposed to problems with either ventricular looping or specific ventriculo-arterial connections.
